# XRCC2 rs3218536 polymorphism decreases the sensitivity of colorectal cancer cells to poly(ADP-ribose) polymerase 1 inhibitor

**DOI:** 10.3892/ol.2014.2236

**Published:** 2014-06-11

**Authors:** KAIWU XU, XINMING SONG, ZHIHUI CHEN, CHANGJIANG QIN, YULONG HE

**Affiliations:** Department of Gastrointestinal and Pancreatic Surgery, The First Affiliated Hospital of Sun Yat-sen University, Guangzhou, Guangdong 510080, P.R. China

**Keywords:** XRCC2, rs3218536 mutation, colorectal cancer, poly(ADP-ribose) polymerase 1 inhibitor

## Abstract

Single nucleotide polymorphisms (SNPs) are associated with the development of certain types of cancer. The present study aimed to investigate the association between X-ray repair complementing defective repair in Chinese hamster cells 2 (XRCC2) SNPs and colorectal cancer (CRC) cell sensitivity to the poly(ADP-ribose) polymerase (PARP) 1 inhibitor olaparib (AZD2281). SNaPshot^®^ analysis of XRCC2 SNPs was performed in five CRC cell lines. The AZD2281-sensitivities of the CRC cells were also analyzed using MTT assays. The effect of AZD2281 on XRCC2 and PARP1 expression was investigated in the five cell lines using quantitative polymerase chain reaction and western blot analyses. Parallel investigations were performed using a cisplatin (DDP) model of DNA damage. The XRCC2 rs3218536 SNP was found to be associated with the LoVo microsatellite instability CRC cell line. The relative rate of growth inhibition was found to be lower in the LoVo cells following treatment with AZD2281 compared with the other four cell lines (P=0.002). Furthermore, the XRCC2 mRNA level in the LoVo cells was observed to be significantly higher than that in the other four cell lines (P<0.05). Similar results were found using the DDP model of DNA damage (P<0.05). The present study indicated that the XRCC2 rs3218536 polymorphism decreases the sensitivity of CRC cells to AZD2281.

## Introduction

Due to germline and epigenetic gene inactivation, tumors with microsatellite instability (MSI) account for ~20% of all types of colorectal cancer (CRC) (1,2). Compared with microsatellite stable (MSS) tumors, sporadic MSI tumors exhibit recognizable clinicopathological features, including the absence of necrosis, the presence of a Crohn’s-like reactions, a right-sided location, a lower pathological stage and an improved prognosis (2–4). Genetic instability in sporadic MSI tumors primarily reflects variation in microsatellite tracts as a result of defective surveillance mechanisms controlled by the DNA mismatch repair system. At present, 30 target genes have been identified to be involved in MSI carcinogenesis, including genes of the homologous recombination repair (HRR) pathway for double-strand breaks (DSBs), such as MRE11, RAD50 and Ku80 (5,6). Thus, other HRR genes, for example, X-ray repair complementing defective repair in Chinese hamster cells 2 (XRCC2) may also be associated with MSI.

Located on chromosome 7q36.1, XRCC2 encodes a member of the Rad51 family of related proteins that maintains chromosome stability by participating in homologous recombination and repairs DNA damage. XRCC2 has roles in the HRR pathway of double-stranded DNA, which repairs chromosomal fragmentation, deletions and translocations (7–9). In total, ~622 single nucleotide polymorphisms (SNPs) have been reported in XRCC2, including rs3218536 (Arg188His), rs718282, rs3218384, rs3218550, rs3218408, rs2040639 and rs3218499. Studies have been performed on the association between SNPs and the cancer incidence risk in ~250,000 individuals from a number of countries, including the United States of America, China, Japan, Poland and Iran, however, the findings have been inconsistent (10–16). However, certain studies have shown that XRCC2 SNPs increase the risk of CRC (16,17).

DSBs are one of the most significant threats to genomic integrity and induce the activation of repair proteins that are involved in the HRR and non-homologous end-joining (NHEJ) pathways; for example, D-NHEJ, which uses DNA-dependent protein kinase, and B-NHEJ, which uses DNA ligase III and poly(ADP-ribose) polymerase (PARP) 1. PARP1 has a key role in HRR and NHEJ. Inhibitors of PARP1 increase the levels of persistent single-strand breaks that lead to DNA DSBs upon replication (18,19). According to the theory of synthetic lethality, cells may survive when either a HRR gene, such as XRCC2, is mutated, or when PARP1 is inhibited, but not when both occur simultaneously (20). The present study aimed to investigate the activity of the PARP1 inhibitor olaparib (AZD2281) in CRC cell lines with XRCC2 SNP mutations.

## Materials and methods

### Cell lines

CRC cell lines (LoVo, LS174T, HT29, SW480 and SW620) were purchased from the American Type Culture Collection (Manassas, VA, USA) and maintained in Dulbecco’s modified Eagle’s medium (Invitrogen Life Technologies, Carlsbad, CA, USA) supplemented with 10% fetal bovine serum (HyClone Laboratories, Inc., Logan, UT, USA). The cells were maintained in a humidified atmosphere at 37°C with 5% CO_2_.

### SNaPshot^®^ analysis of SNP genotypes

SNaPshot analysis of SNP genotypes was performed using a SNaPshot Multiplex kit system (Applied Biosystems, Foster City, CA, USA). Products were treated with 1 unit of shrimp alkaline phosphatase at 37°C for 60 min and 75°C for 15 min, followed by a denaturation step at 95°C for 5 min. Detection was performed using 0.5 μl SNaPshot products mixed with 9 μl HiDi™ formamide and 0.5 μl GeneScan-120LIZ size standard (Applied Biosystems). Data were generated following capillary electrophoresis on an automated sequencer (ABI 3130 Genetic Analyzer; Applied Biosystems) with a 36-cm length capillary and POP-7™ polymer and analyzed using GeneMapper^®^ software version 3.7 (Applied Biosystems).

### MTT assay

MTT assays were performed to quantify the viability of the CRC cells following treatment with the PARP1 inhibitor, AZD2281, and cisplatin (DDP). MTT stock solution (0.5 mg/ml; Sigma-Aldrich, St. Louis, MO, USA) was added to the incubation medium in the wells at a final concentration of 25 μmol/l AZD2281 (Selleckchem, Houston, TX, USA) and 0.75 μg/ml DDP (Nuoxin; Jiangsu Hansoh Pharmaceutical Co., Ltd., Group, Lianyungang, China). The cells were incubated for 36 h at 37°C in a humidified 5% CO_2_ atmosphere. The culture medium was then removed and the formazan crystals in the cells were solubilized using dimethyl sulfoxide (Sigma-Aldrich), with plate agitation for 30 min. The absorbance was measured at 570 nm, with a reference wavelength of 655 nm.

### Expression of XRCC2 wild-type and mutant transcripts and PARP1

Total RNA was extracted from the cultured cells using TRIzol^®^ reagent (Invitrogen Life Technologies) according to the manufacturer’s instructions. The cDNA was amplified and quantified using an ABI Prism 7500 Sequence Detection System (Applied Biosystems) with SYBR Green I dye (Invitrogen Life Technologies). The following primers were used: XRCC2 forward, TCACCTGTGCATGGTGATATT and reverse, TTCCAGGCCACCTTCTGATT; PARP1 forward, ACAGTGTGCAGGCCAAGGTG and reverse, CTCGGCTTCTTCAGAATCTCTGTC; β-actin forward, TGGCACCCAGCACAATGAA and reverse, CTAAGTCATAGTCCGCCTAGAAGCA. Expression data were normalized to the expression of β-actin and calculated as 2^− [(Ct of gene) − (Ct of β-Actin)]^, where Ct represents the threshold cycle for each transcript.

### Western blot analysis

The cells were harvested in sampling buffer [62.5 mmol/l Tris-HCl (pH 6.8), 10% glycerol and 2% SDS] and heated for 5 min at 100°C. The protein concentration was determined using the Bradford assay using a commercial kit purchased from Bio-Rad Laboratories, Inc. (Hercules, CA, USA). Equal quantities of protein were separated electrophoretically on 12% SDS polyacrylamide gels and transferred onto polyvinylidene difluoride membranes (Roche, Basel, Switzerland). The membranes were probed for 3 h with mouse anti-XRCC2 (dilution, 1:1,000; ab20253; Abcam Plc, Cambridge, UK) and mouse anti-PARP1 (dilution, 1:1,000; ab96476; Abcam Plc) antibodies. The expression of the target proteins was determined using horseradish peroxidase-conjugated anti-rabbit/anti-mouse immunoglobulin G (dilution, 1:3,000) and enhanced chemiluminescence (Pierce Chemical Company, Rockford, IL, USA) according to the manufacturer’s instructions. The membranes were stripped and reprobed with an anti-β-actin mouse monoclonal antibody (dilution, 1:1,000; Sigma-Aldrich) as a loading control.

### Statistical analysis

Differences among the cell lines were analyzed following treatment with AZD2281 and DDP using repeated measures analysis of variance (ANOVA), covariance analysis and pairwise comparison methods. The two-tailed Student’s t-test was used to assess the significance of the differences between two groups of data. P<0.05 was considered to indicate a statistically significant difference.

## Results

### XRCC2 rs3218536 polymorphism mutation is associated with MSI CRC cell lines

**Figure 1:**
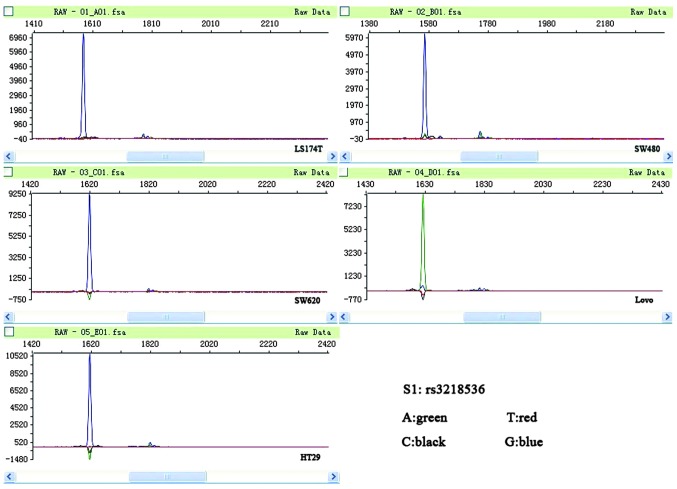
Peak figure of XRCC2 rs3218536. A green peak, representing A, is apparent in the microsatellite instability (MSI) LoVo cell line, and blue peaks, representing G, are apparent in the other cell lines. A, adenine; T, thymine; C, cytosine; G, guanine; XRCC2, X-ray repair complementing defective repair in Chinese hamster cells 2.

LoVo and LS174T are MSI cell lines, while SW480, SW620 and HT29 are MSS cell lines (21). In the present study, mutations in the coding microsatellite tracts of XRCC2 were assessed in a panel of five CRC cell lines. All the MSI cell lines were found to have a mutation in XRCC2, while none of the MSS cell lines were found to have a mutation (P=0.025). The two MSI cell lines (LoVo and LS174T) were observed to have mutations in the poly-(A) tract located on exon 3 of XRCC2; the rs3218536 mutation was homozygous, while the rs3218550 mutation was heterozygous. The rs3218550 mutation was not located within a coding DNA sequence (CDS); therefore, the present study focused on the rs3218536 mutation. None of the MSS cell lines had mutations in XRCC2 (Fig. 1; Table I).

### Low AZD2281-sensitivity of MSI LoVo cells

The five cell lines were divided into three groups: Group 1, LoVo cells that harbored a mutation in the poly-(A) tract of XRCC2; group 2, LS174T cells that harbored another mutation not located within a CDS region; and group 3 (control), SW480, SW620 and HT29 cells that did not harbor mutations in the poly-(A) tract of XRCC2.

**Figure 2:**
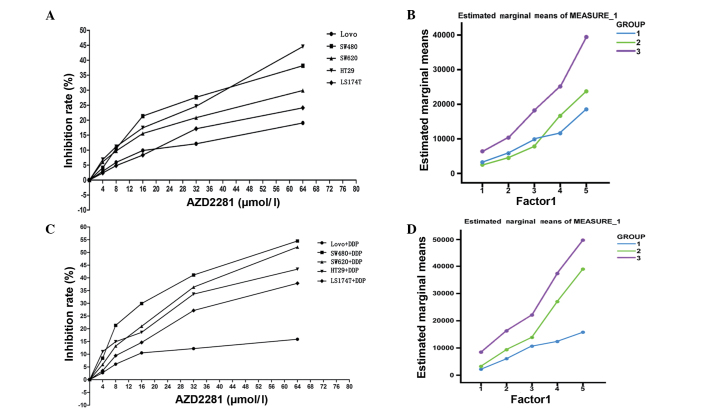
(A) Relative rate of growth inhibition to AZD2281 in the five cell lines measured using MTT assay. (B) Estimated marginal means of the three groups following treatment with AZD2281. (C) Relative inhibition rates of the five cell lines in response to treatment with AZD2281 and DDP. (D) The estimated marginal means of the three groups in response to treatment with AZD2281 and DDP. DDP, cisplatin; AZD2281, olaparib.

The relative growth inhibition rate of the cell lines mediated by different concentrations of the novel PARP1 inhibitor, AZD2281, was analyzed. The half maximal inhibitory concentration (IC_50_) in the cells in group 1 (1797.2 μmol/l) was approximately three-fold higher than that in the cells in the other two groups (group 2, 631.7 μmol/l; group 3, 579.58 μmol/l). The relative inhibition rate of the cells was observed to be affected by the AZD2281 concentration (0–64 μmol/l; P<0.001). Furthermore, the relative inhibition rate of the LoVo cells in response to AZD2281 was found to be significantly lower than that of the other cell lines (P=0.002) (Fig. 2A; Table II). There was no significant difference in the estimated marginal means of the cells in group 1 and group 2 (P=0.415), while the estimated marginal mean of the cells in group 1 was significantly lower than that of group 3 (P<0.001; Fig. 2B).

According to repeated measures ANOVA, the relative inhibition rate of the cells was affected by the concentration of AZD2281 and DDP (P<0.001) and the defining group factors (P<0.001). Furthermore, there were cross-effects between the different concentrations of AZD2281 and DDP and the defining group factors (P<0.001; Fig. 2C; Table III). The estimated marginal mean of group 1 was found to be lower than that of group 2 (P=0.004) and group 3 (P<0.001; Fig. 2D).

### PARP1 expression is not associated with the XRCC2 rs3218536 mutation

**Figure 3:**
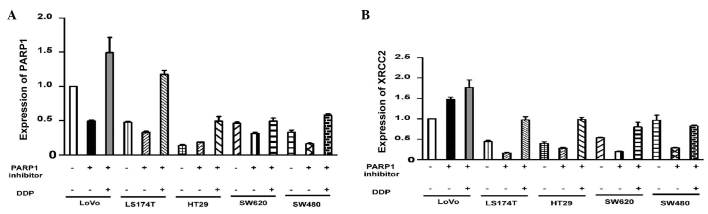
(A) PARP1 and (B) XRCC2 mRNA expression in the five cell lines following treatment with AZD2281 and DDP. PARP, poly(ADP-ribose) polymerase; XRCC2, X-ray repair complementing defective repair in Chinese hamster cells 2; DDP, cisplatin; AZD2281, olaparib.

**Figure 4:**
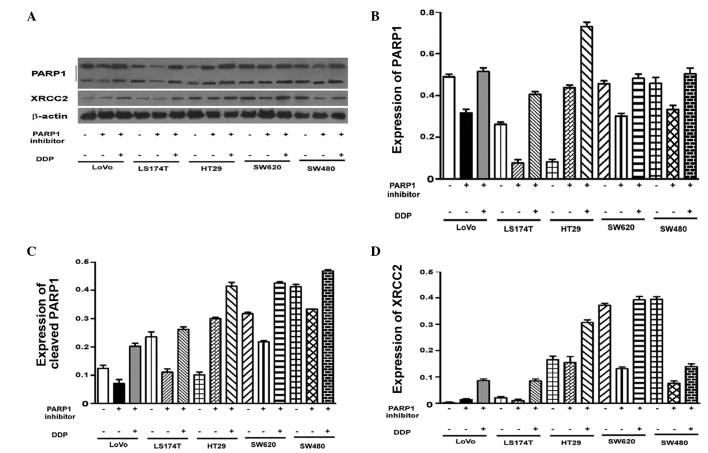
(A) Western blot analysis of PARP1 and XRCC2 protein expression in the five cell lines in response to treatment with AZD2281 and DDP. Protein expression of (B) PARP1 (C) cleaved PARP1 and (D) XRCC2 following treatment with AZD2281 and DDP. PARP, poly(ADP-ribose) polymerase; XRCC2, X-ray repair complementing defective repair in Chinese hamster cells 2; DDP, cisplatin; AZD2281, olaparib.

Varying levels of PARP1 expression were observed in the untreated cell lines (P<0.001; Fig. 3A). To assess the effect of AZD2281 and DDP on the expression of PARP1, the mRNA levels in each cell line were adjusted to the same level. Pairwise comparisons of the PARP1 mRNA levels in the cells revealed that there were no significant differences in PARP1 mRNA expression in the LoVo cells compared with the other four cell lines following treatment with AZD2281 and DDP (P>0.05; Table IV). Western blot analysis demonstrated that there were no significant differences in PARP1 protein expression between the cell lines (Fig. 4). Thus, the effect of AZD2281 and DDP on PARP1 mRNA levels was unaffected by the original PARP1 mRNA levels (P=0.835; P>0.05).

### High XRCC2 expression in LoVo cells

Following AZD2281 treatment, XRCC2 expression was found to be associated with the cell group (P<0.001). The LoVo cells had significantly higher XRCC2 mRNA levels compared with the other four cell lines (P<0.001). Notably, XRCC2 expression following AZD2281 and DDP treatment, was also closely associated with the cell group (P<0.001). The level of XRCC2 mRNA expression in the LoVo cells was significantly higher than that in the other four cell lines (P<0.001; Fig. 3B; Table V). The results of the western blot analysis were consistent with the results of the quantitative polymerase chain reaction analysis (Fig. 4A and D).

## Discussion

Simultaneous deficiencies in two genes have been proposed to introduce lethality in biologic systems that would otherwise tolerate the loss of one of the genes (22). According to this theory, the inhibition of PARP is a potential therapeutic strategy for the treatment of types of cancer that have specific DNA repair defects, including those that arise in individuals who are carriers of mutations in BRCA1 or BRCA2 (23). Other important genes involved in HRR include MRE11, XRCC2 and PARP1. In the present study, it was hypothesized that other components of this pathway may predict an increase in PARP1 inhibitor sensitivity. XRCC2 has roles in the HRR pathway, which repairs chromosomal fragmentation, deletions and translocations. Numerous studies have reported an association between XRCC2 SNPs and the cancer incidence risk; the most common mutation studied being the XRCC2 rs3218536 SNP (10–17). The present study aimed to investigate the effect of this SNP on PARP1 inhibitor sensitivity in CRC cell lines.

Vilar *et al* (21) reported that LoVo and LS174T are MSI cell lines that have biallelic mutations in MRE11. However, the study reported that there were no such mutations in MSS cell lines, including the SW480, SW620 and HT29 cell lines (21). In the present study, the rs3218536 and rs3218550 SNP mutations of XRCC2 were only found to occur in two MSI cells. In the MSS cell lines, no SNP mutations were observed in XRCC2. However, since only five CRC cell lines were investigated in the present study, whether these SNP mutations occur only in MSI CRC lines remains to be investigated.

Certain PARP1 inhibitors, including benzimidazole-4-carboxamides and tricyclic lactam indoles, have been reported to inhibit cell growth by 50% at concentrations between 8 and 94 μM (24). Vilar *et a*l (21) found that following treatment with the PARP1 inhibitor veliparib (ABT-888) at 10 μM, there was a significant difference in cytotoxicity between biallelic mutants cell lines, such as LoVo, and wild-type cell lines, such as SW480 (P=0.028), with a 2.5-fold difference in IC_50_ between these two groups (P=0.028). The study concluded that cell lines, such as LoVo, that harbor biallelic mutations in MRE11, have a higher sensitivity compared with wild-type cell lines, such as SW480 (21). In the present study, LoVo cells were found to harbor the XRCC2 rs3218536 mutation and were considered to be a SNP mutation-positive cell line compared with the other cell lines investigated, which were regarded as control cell lines. According to the theory of synthetic lethality and the findings of Vilar *et al* (21), LoVo cells should have a higher sensitivity to the PARP1 inhibitor AZD2281, as well as have a correspondingly lower IC_50_ compared with the other four cell lines, due to the presence of the XRCC2 rs3218536 mutation. In accordance with this, the relative growth inhibition rate of the LoVo cells should be higher than that of the other four cell lines in response to AZD2281. The results of the present study were contrary to this expectation. In the present study, the LoVo cells exhibited a lower sensitivity to the PARP1 inhibitor, AZD2281, and a higher IC_50_ compared with the other four cell lines. The differences in the results reported in the present study compared with those reported by Vilar *et al* (21) may be due to differences in the PARP1 inhibitors used.

In the present study, no differences in PARP1 mRNA levels were detected in the LoVo cells compared with the other four cell lines. Furthermore, a higher level of XRCC2 mRNA was detected in the LoVo cells compared with the other four cell lines. These findings indicate that higher levels of XRCC2 mRNA are associated with a lower sensitivity to the PARP1 inhibitor, AZD2281. Moreover, consistent results were found in the analysis of the effect of the PARP1 inhibitor on the five cell lines in a DNA damage model based on DDP treatment for 16–32 h. These results are consistent with those of Vilar *et al* (21).

In conclusion, the results of the present study show that there is no association between PARP1 inhibitor sensitivity and XRCC2 SNP mutations in CRC cells. However, due to the limitations of the study, this conclusion requires further investigation to fully elucidate this association. To the best of our knowledge, there are >600 SNP sites in XRCC2, seven of which have been reported in previous studies (10–17). The rs3218536 SNP is located in a CDS region, therefore, it is possible that this SNP mutation leads to a decreased sensitivity to AZD2281, while other SNPs may not. Furthermore, a number of types of PARP1 inhibitors are available, which may yield different results in similar studies. Moreover, AZD2281 may not have an effect on the DNA damage induced by DDP. These factors will be clarified in a future rs3218536 mutation model that will be developed to facilitate further investigations.

## Figures and Tables

**Table I:** Primer sequences used for multiplex polymerase chain reaction amplification panels.

Gene	SNP	Forward (5′-3′)	Reverse (5′-3′)	Amplicon size, bp
XRCC2	rs3218536	TGTAGTCACCCATCTCTCTGC	CACAGTCGTCGAGAGGCATGA	240
	rs718282	GATACTTGGGAGATTGAGGCA	GCCTGCTGTTATGAGTGTGAA	205
	rs3218384	ACACCCTATTGCGCATGCTCC	CCCATCTCCCTCACTCCCAAC	233
	rs3218550	CATTCAACCCAGCAATCTCAT	CACGCCCAGTCAGTCTTGTT	190
	rs2040639	ATGCCTACCAGCAGTTTGTGA	CAGTCTCCACACTGTTCCTAATG	183
	rs3218499	ATATCTTCAAGTGCCAAACCT	GATCCTCAAGATCAAAACCTG	186
	rs3218408	TAGGCGATATACTGATGCCCT	CAAGGCATGCACAGGCAGAAC	190

SNP, single nucleotide polymorphism; XRCC2, X-ray repair complementing defective repair in Chinese hamster cells 2.

**Table II:** Repeated measures analysis of variance of cells following treatment with AZD2281.

				95% confidence interval	
				
Group	Mean	Std	Pairwise comparisons P-value	Lower bound	Upper bound
1	9.967	1.157		7.447	12.487
2	11.349	1.157	0.415	8.828	13.869
3	19.221	0.668	<0.001	17.766	20.676

Std, standard error; AZD2281, olaparib.

**Table III:** Repeated measures analysis of variance of cells following treatment with AZD2281and DDP.

				95% confidence interval	
				
Group	Mean	Std	Pairwise comparisons P-value	Lower bound	Upper bound
1	9.482	1.795		5.571	13.393
2	18.505	1.795	0.004	14.594	22.416
3	26.986	1.036	<0.001	24.728	29.244

Std, standard error; DDP, cisplatin; AZD2281, olaparib.

**Table IV:** Pairwise comparisons of PARP1 mRNA expression between different cell lines.

				95% confidence interval	
				
Group	Mean	Standard error	Pairwise comparisons P-value	Lower bound	Upper bound
LoVo	0.429	0.152		0.086	0.773
LS174T	0.329	0.009	0.527	0.308	0.349
HT29	0.227	0.101	0.445	−0.002	0.457
SW480	0.183	0.045	0.240	0.082	0.284
SW620	0.317	0.010	0.493	0.293	0.341

PARP, poly(ADP-ribose) polymerase.

**Table V:** Pairwise comparisons of XRCC2 mRNA expression of cells.

				95% confidence interval	
				
Group	Mean	Standard error	Pairwise comparisons P-value	Lower bound	Upper bound
LoVo	1.470	0.055		1.346	1.594
LS174T	0.168	0.040	<0.001	0.078	0.257
HT29	0.286	0.046	<0.001	0.182	0.390
SW480	0.285	0.050	<0.001	0.172	0.397
SW620	0.202	0.027	<0.001	0.142	0.263

XRCC2, X-ray repair complementing defective repair in Chinese hamster cells 2.
